# Pricing decisions of converted and expanded enterprises for medical supplies under major public health emergencies

**DOI:** 10.3389/fpubh.2026.1831818

**Published:** 2026-06-09

**Authors:** Lijing Du, Meili Fan, Yong Lin

**Affiliations:** 1 School of Management, Wuhan University of Technology, Wuhan, China; 2 Birmingham Business School, University of Birmingham, Birmingham, United Kingdom

**Keywords:** enterprise economics, government subsidies, major public health emergencies, pricing decisions, Stackelberg game

## Abstract

**Background:**

Major public health emergencies often trigger severe imbalances between the supply and demand of medical supplies, resulting in substantial price fluctuations and reduced allocation efficiency. The rapid entry of converted and capacity-expanding enterprises into medical supply production further complicates pricing dynamics, particularly under government intervention policies.

**Methods:**

To investigate optimal pricing decisions under emergency conditions, this study integrates an epidemiological SEIR framework with an economic decision model and develops a Stackelberg game involving converted and expanded manufacturers under government subsidy schemes. Pricing strategies are compared under decentralized competitive and centralized cooperative scenarios to evaluate differences in market outcomes and welfare performance.

**Results:**

The data suggest that decentralized competition among businesses results in inflated prices and diminished efficiency in resource allocation. Conversely, collaborative pricing and resource integration demonstrably enhance firm profitability and contribute to greater overall social welfare. Furthermore, when production costs rise for manufacturers that have undergone conversion, the adverse impacts of cost escalation can be mitigated through the redistribution of demand among enterprises.

**Conclusion:**

This research furnishes a theoretical basis for pricing strategies concerning medical supplies during significant public health crises. The findings offer practical guidance for policymakers in the formulation of effective subsidy mechanisms and price regulation policies, thereby fortifying emergency medical supply systems. This study provides a theoretical foundation for pricing strategies of medical supplies during major public health emergencies. The results offer practical insights for policymakers in designing effective subsidy mechanisms and price regulation policies, thereby strengthening emergency medical supply.

## 1 Introduction

Major public health emergency refers to sudden outbreaks of infectious diseases or other events that cause significant harm to public health and social operations within a short period, characterized by rapid transmission, wide-ranging impact, and strong external influence. The COVID-19 pandemic that emerged in 2020 caused massive infections worldwide, severely disrupting social production and daily life (1). Ensuring the supply of consumable medical supplies such as masks, protective clothing, and vaccines becomes crucial during major public health emergencies (2). When such events occur, the demand for medical supplies surges dramatically. However, shortages and price increases in medical supplies arise due to multiple factors including raw material constraints and transportation restrictions (3, 4). During the 2019 major public health emergency, the supply chain for consumable medical supplies such as masks involved both incumbent expansion enterprises and emergency transition enterprises that repurposed production in response to urgent shortages (5, 6). Expansion enterprises typically possess mature technical reserves, production experience, and distribution networks, giving them a first-mover advantage in capacity expansion. Transition enterprises, driven by emergency needs, enter the market with varying production efficiency, quality stability, and demand awareness compared to expansion enterprises (7). Competition may coexist with cooperation through pricing, output, and investment decisions. To alleviate the supply–demand imbalance, China implemented policies to boost production and supply, including fiscal subsidies to encourage enterprises to transition and expand medical supplies (8).

Supported by policy incentives and cross-sector industrial coordination, medical supply production capacity expanded markedly during the COVID-19 pandemic. In China, the daily output of non-N95 medical masks rose from several million units in early February 2020 to over 200 million units by late April, indicating the effectiveness of emergency capacity mobilization (9). However, in the context of major public health emergencies, substantial demand uncertainty and the limited responsiveness of production planning may lead to pronounced fluctuations in the prices of essential medical supplies. In order to contain excessive price increases arising from market distortions, governments commonly adopt regulatory interventions, including price controls on critical products such as masks (3).

Rising raw material costs, procurement constraints, and emergency operating expenses may weaken production incentives during major public health emergencies (4). At the same time, unusual purchasing behavior such as stockpiling and panic buying can further aggravate shortages and intensify price pressure in medical supply markets (10, 11). For enterprises transitioning into medical supply production, low regulated prices may be insufficient to cover the additional costs of equipment adjustment, raw material sourcing, and emergency operations, thereby reducing their willingness to sustain production (4). In addition, perceived quality and consumer trust may differ across producers, which adds further complexity to pricing decisions for medical supplies under emergency conditions (12).

Recent studies on epidemic-related economic decision-making have made important progress in explaining supply chain disruption, demand uncertainty, emergency procurement, medical supply reserves, government subsidies, and coordination mechanisms during public health crises. Li and Dong examined how government regulations can mitigate shortages of life-saving goods during a pandemic and showed that policy intervention plays an important role in stabilizing emergency markets (3). Huang et al. studied optimal reserve policies for emergency medical supplies by jointly considering safety stocks, production capacity, and capital constraints, highlighting the importance of integrated reserve planning (13). Yang et al. (14) investigated subsidy strategies for reserving flexible emergency production capacity and demonstrated that government support can influence firms’ willingness to maintain emergency supply capability. Zhou et al. (15) further developed a multi-stage public-private cooperation framework for emergency supply reserves and emphasized the role of enterprise participation, reputation, and government coordination in improving emergency preparedness. These studies provide valuable insights into how epidemics affect emergency supply chains and how policy interventions may reduce shortages. However, the position of firm-level strategic behavior within epidemic-economic modeling remains insufficiently developed. In many SEIR-based studies, the epidemic process is modeled in detail, but enterprise production, pricing, and subsidy responses are usually simplified or treated as external factors. In contrast, supply chain and game-theoretic studies often analyze pricing, subsidies, contracts, and coordination, but epidemic effects are commonly represented by exogenous demand shocks, stochastic demand, or background emergency scenarios. As a result, the mechanism through which infection prevalence generates medical supply demand and further affects enterprise pricing and welfare outcomes has not been fully explained.

The novelty of this study lies in constructing a coupled SEIR–Stackelberg framework rather than applying the two models independently. In this framework, the SEIR model provides a dynamic epidemiological basis for medical supply demand: the number of infectious individuals is used to represent the intensity of public health pressure and is further transformed into time-varying demand for medical protective supplies. The Stackelberg model then embeds this epidemic-driven demand into the strategic decision-making process of heterogeneous enterprises. Specifically, capacity-expanding enterprises, which have stronger production experience and lower marginal costs, are modeled as market leaders, whereas converted enterprises, which enter the market under emergency conditions, are modeled as followers. Government subsidies further affect the cost–benefit structure of both types of enterprises.

In summary, the core of medical supply management lies in establishing pricing strategies for enterprises transitioning or expanding production of medical supplies during major public health emergencies, based on government subsidy policies and considering the cost–benefit analysis of medical supplies and quality perception differences (15, 16). As a common protective measure to block respiratory infectious disease transmission, masks were widely used in public health events such as SARS, H1N1, and Ebola, and remain essential for COVID-19 prevention and control (17–19). This study takes masks as a representative medical supply, calculates medical supply demand based on the evolution of major public health emergencies, and compares pricing and supply behavior decisions of enterprises transitioning or expanding production under two scenarios competition and cooperation, based on government subsidies. The findings provide references for policy design and collaborative governance in emergency medical supply production and price control.

## 2 Literature review

Compartmental models are among the most widely used approaches in infectious disease research. These models divide the population into compartments according to epidemiological status and describe the dynamic transitions between compartments using systems of differential equations, thereby characterizing the spread of infectious diseases (20). The SEIR model (Susceptible-Exposed-Infectious-Removed) is an improved compartmental model widely used in the field of epidemic evolution (21). For major public health emergencies, Reiner et al. (22) used a deterministic SEIR framework to model possible trajectories of COVID-19 infections in the United States and to assess the effects of non-pharmaceutical interventions. Kumar et al. (23) further developed a modified SEIR-V model with an additional social media component to investigate how social media influences influenza and COVID-19 infections and deaths. In studies on the spatial organization of epidemic prevention resources, recent research has examined the spatial distribution of medical facilities and their relationship with epidemic response capacity in Chinese cities, showing that differences in medical resource layout may affect accessibility and emergency response performance (24, 25). In terms of transmission-mechanism modeling, Qian and Ukkusuri proposed the Trans-SEIR framework, which incorporates travel demand and daily activity patterns into epidemic transmission analysis, thereby providing a more realistic account of disease spread in urban environments (26). In addition, some studies have incorporated population mobility into SEIR-based frameworks to improve the prediction of COVID-19 epidemic trends. For example, Ye et al. (27) combined mobility information and epidemic prevention measures with a mathematical modeling framework to forecast the evolution of COVID-19 in Yueqing, Zhejiang Province. More broadly, infectious disease models have been widely used to simulate epidemic transmission processes, evaluate intervention effects, and provide quantitative support for epidemic prevention and control strategies (28). Under pandemic uncertainty, emergency procurement and allocation decisions are better treated as a rolling process, in which plans are revised phase by phase as updated information on demand and disruptions becomes available (29). Data-driven decision support can further enhance the robustness and responsiveness of such emergency operations (30). Overall, SEIR-based models and their extensions have become widely used tools for describing epidemic transmission dynamics. They are also increasingly applied to forecasting healthcare demand, including hospital admissions and bed occupancy, thereby providing quantitative support for epidemic preparedness and resource planning (31, 32).

In major public health emergencies, the demand for medical supplies often rises sharply within a short period, whereas supply is mainly replenished through emergency reserves, production capacity reserves, and firms’ decisions to switch or expand production under government support (13, 14, 33). Existing studies have further shown that, under enterprise-based reserve arrangements, reserve decisions are sensitive to procurement pricing, emergency occurrence risk, and subsidy design (33, 34). These factors jointly shape firms’ willingness to participate in reserve programs and their preferred reserve strategies. Angelus and Özer (35) developed a stochastic, multi-period sequential decision model to examine how production organization and capacity expansion strategies affect large-scale vaccine manufacturing decisions. Their study provides insights into the optimal arrangement of vaccine production capacity under uncertainty. In a related stream of research, Sun et al. investigated at-risk production capacity building for COVID-19 vaccines and showed that capacity investment is shaped by the incentive alignment between firms and public authorities, with important implications for the design of policy support and capacity-expansion mechanisms (36) Recent studies have examined how medical supply production can be rapidly reconfigured during public health emergencies. Using ventilators as an example, Malik et al. (37) explored how production systems can be reorganized and ramped up under epidemic conditions, with particular attention to flexible assembly arrangements and rapid capacity scaling. Gambaro et al. (38) further developed a dynamic decision framework for resource expansion in the early stage of a pandemic, linking epidemic uncertainty with capacity expansion decisions and highlighting the importance of timing in resource deployment. Related case-based research has also documented how manufacturing firms temporarily shifted production to medical protective products during COVID-19, illustrating the operational and certification challenges involved in emergency repurposing (39). In practice, governments supported the recovery and expansion of medical supply production through a combination of tax and fee reductions, fiscal support, and policies aimed at accelerating the resumption and expansion of epidemic-prevention material production (40).

Taken together, existing studies provide important foundations for understanding epidemic transmission, emergency supply management, capacity expansion, and government intervention. However, these research streams have largely developed along related but still partially separated lines. SEIR-based and mobility-based models improve the understanding of infection dynamics, but they usually do not explain how enterprises adjust pricing and production strategies in response to epidemic-induced demand. Emergency procurement and capacity expansion studies offer useful insights into supply preparedness and production adjustment, but demand is often treated as uncertain or externally given. Game-theoretic studies are effective in analyzing subsidies, contracts, and coordination, but many of them do not derive market demand from the epidemic process itself.

As shown in Table 1, the main gap in the existing literature lies in the insufficient integration of epidemic-driven demand formation, enterprise heterogeneity, strategic pricing, and welfare evaluation. This study addresses this gap by constructing a coupled SEIR–Stackelberg framework. In this framework, infection prevalence generates time-varying demand for medical protective supplies, while heterogeneous enterprises make pricing decisions under government subsidy intervention. By comparing competitive and cooperative decision-making modes, this study explains how epidemic pressure and policy intervention are transmitted into prices, profits, consumer surplus, and social welfare. This integrated approach distinguishes the present study from conventional epidemic modeling, emergency supply chain research, and broader epidemic-economic integration studies, and provides a more comprehensive basis for emergency medical supply policy design.

**Table 1:** Comparison between existing research streams and this study.

Research stream	Typical focus	Main gap	This study
SEIR-based epidemic models	Transmission dynamics and intervention effects	Limited treatment of firm decisions	Converts infection dynamics into medical supply demand
Emergency supply studies	Procurement, reserves, and capacity expansion	Demand is often exogenous	Links demand to epidemic evolution
Supply chain game models	Pricing, subsidies, and coordination	Weak connection with epidemic mechanisms	Embeds epidemic-driven demand into a Stackelberg game
Epidemic-economic models	Epidemic shocks and economic outcomes	Limited firm-level strategic mechanism	Connects infection evolution, enterprise heterogeneity, subsidies, and welfare
This study	Medical supply pricing	–	Provides an integrated SEIR–Stackelberg framework for competition and cooperation scenarios

## 3 Model formulation

To characterize the dynamic evolution of medical supply demand during major public health emergencies and to investigate how government subsidy policies influence enterprise production adjustment decisions, this study develops an integrated analytical framework that combines epidemiological modeling with economic decision analysis. Specifically, an SEIR-based epidemic transmission model is first employed to capture the progression of disease spread and its impact on the demand for medical supplies. Government subsidy mechanisms are then incorporated into the analytical structure, and a Stackelberg game model involving converted and capacity-expanding enterprises is constructed. Within this framework, the optimal production and pricing decisions of enterprises under different production modes are systematically examined.

### 3.1 SEIR epidemic transmission model

To describe the impact of epidemic transmission on medical supply demand, the classical Susceptible–Infectious–Removed (SIR) model is extended by incorporating an exposed population compartment. Considering the preventive effect of mask-wearing behavior on transmission dynamics, a modified SEIR model is developed to better reflect realistic epidemic characteristics and complex public health conditions.

The epidemic transmission process is characterized by the following system of ordinary differential (Equation 1):


$$\begin{array}{c} \frac{\mathrm{dS}}{\mathrm{dt}}=-[\beta ]\frac{S(t)I(t)}{N}\\ \frac{\mathrm{dE}}{\mathrm{dt}}=[\beta ]\frac{S(t)I(t)}{N(t)}-\mathrm{\sigma E}(t)\\ \frac{\mathrm{dI}}{\mathrm{dt}}=\mathrm{\sigma E}(t)-\mathrm{\mu I}(t)-\mathrm{\delta I}(t)\\ \frac{\mathrm{dR}}{\mathrm{dt}}=\mathrm{\mu I}(t) \end{array}$$ (1)

**Figure 1:**
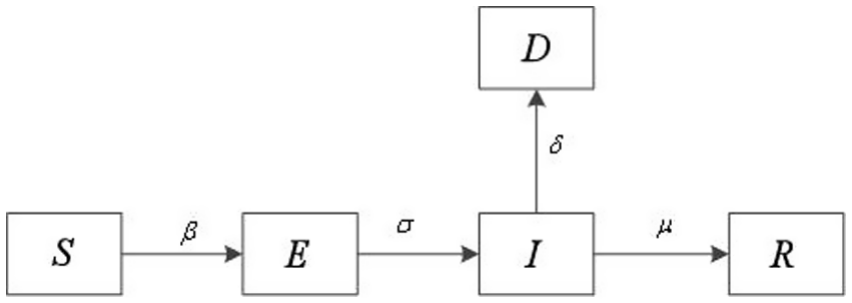
SEIR transmission dynamics model.

To ensure the interpretability and solvability of the model, this study makes the following assumptions:

1. The total population in the study area is divided into four epidemiological compartments: susceptible individuals S(t) , exposed individuals E(t) , infectious individuals I(t) , and recovered individuals R(t) . The population transitions among these compartments according to the SEIR transmission mechanism. Specifically, susceptible individuals enter the exposed state after effective contact with infectious individuals; exposed individuals become infectious after a certain incubation period; and infectious individuals eventually recover and enter the recovered compartment.
2. The total population size remains constant during the study period. Natural births, natural deaths, and long-term population migration are not considered. Therefore, the total population size satisfies: N=S(t)+E(t)+I(t)+R(t) .
3. Individuals are assumed to follow a homogeneous mixing pattern, meaning that individuals have relatively equal opportunities for contact. Although real populations differ in age, occupation, spatial location, and mobility, this study focuses on the impact of epidemic transmission on medical protective supply demand and enterprise supply decisions. Therefore, the homogeneous mixing assumption is adopted to reduce model complexity. The detailed transmission and transition mechanisms are illustrated in Figure 1.
4. The effective transmission rate β , the incubation transition rate σ , and the recovery rate γ are set as baseline parameters. Among them, 1/σ represents the average incubation period, and 1/γ represents the average infectious period. This setting captures the basic dynamic process of epidemic transmission and provides a foundation for constructing the subsequent demand function.
5. Recovered individuals are assumed to acquire long-term immunity during the study period and do not return to the susceptible state. Therefore, reinfection is not considered in this study. This assumption is suitable for short-term public health emergency scenarios and helps maintain the simplicity and interpretability of the model structure.
6. During epidemic outbreaks, the demand for medical protective supplies is strongly associated with the scale of infection. To quantify the direct relationship between infection prevalence and medical supply demand, the total demand for consumable medical supplies (e.g., masks) is assumed to be positively correlated with the number of infectious individuals and is represented in linear form as: Q(t)=I(t) . Considering real-world factors such as information dissemination, panic-driven purchasing behavior, and government interventions that may amplify or suppress market demand, a proportional adjustment parameter ε is introduced to capture the relationship between predicted medical demand Q(t) and actual market demand D(t) . The total realized market demand is therefore defined as: $D(t)=Q_{0}+\mathrm{\varepsilon Q}(t)=Q_{0}+\mathrm{\varepsilon I}(t)$ .
7. The supply chain decision-making process follows a Stackelberg game structure. The government or the dominant enterprise acts as the leader and makes decisions first, while ordinary enterprises or followers make optimal responses based on the leader’s strategy. This setting reflects the hierarchical relationship among policy guidance, enterprise response, and supply chain coordination in the context of a public health emergency.
8. Government subsidies can influence enterprise supply behavior. Subsidy policies change the cost–benefit structure of enterprises, enhance their willingness to produce and invest in protection, and further affect the supply of medical protective materials and the epidemic transmission outcome. It should be noted that the above assumptions inevitably simplify the real-world epidemic and market environment. Actual epidemic transmission may be affected by spatial mobility, age structure, heterogeneous contact networks, behavioral responses, testing intensity, and policy changes. Similarly, actual medical supply demand may be influenced by household stockpiling, institutional procurement, price expectations, and media information. However, incorporating all these factors would make the model less analytically tractable and obscure the main mechanism examined in this study. The purpose of the SEIR component is to generate a theoretically grounded measure of epidemic pressure, while the purpose of the Stackelberg component is to analyze how heterogeneous enterprises and government subsidies respond to the resulting demand. Therefore, the simplified assumptions are appropriate for identifying the core interaction between epidemic-driven demand and emergency medical supply pricing decisions. The robustness of the conclusions is further examined through numerical sensitivity analysis and Monte Carlo simulation.

### 3.2 Production decision model for converted and capacity-expanding enterprises

This study considers a duopolistic market consisting of one capacity-expanding enterprise (indexed by *e*) and one converted enterprise (indexed by *c*). To encourage increased production of medical supplies, the government provides a per-unit subsidy. Given that capacity-expanding enterprises typically possess more mature production technologies and lower marginal costs, they are assumed to act as market leaders. Converted enterprises, which adjust production lines temporarily, are treated as followers. The interaction between the two firms is therefore modeled as a Stackelberg game to examine production decisions in the medical mask market.

**Figure 2:**
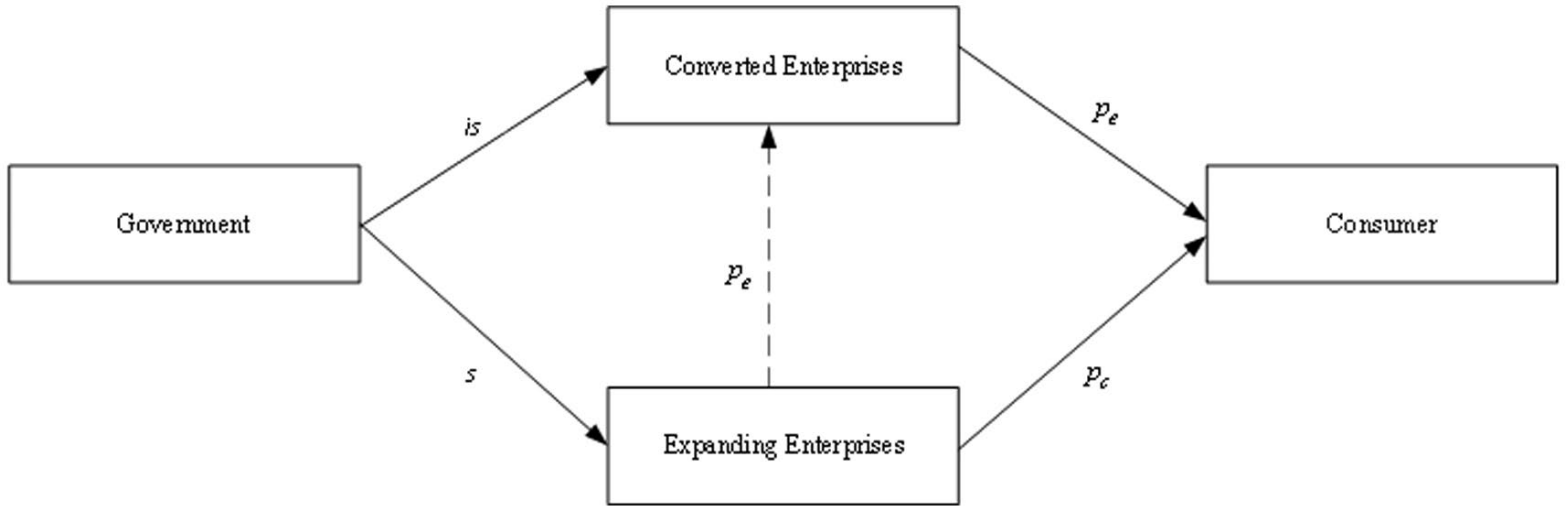
Decision framework of converted and capacity-expanding enterprises under government subsidies.

A detailed description of model notations is provided in the corresponding Table 2, and the decision-making structure is illustrated in Figure 2. In this framework, the capacity-expanding enterprise plays a dominant role in determining market prices.

**Table 2:** Notation table.

Symbol	Description	Economic meaning
k	Cost coefficient of the converted enterprise relative to the capacity-expanding enterprise	Captures the additional cost caused by production conversion, technical adaptation, and operational uncertainty.
i	Subsidy coefficient of the converted enterprise relative to the capacity-expanding enterprise	Measures whether subsidies are tilted toward converted enterprises relative to capacity-expanding enterprises.
ν	Consumers’ willingness to pay	Represents heterogeneous consumer valuation of masks.
$p_{e}$	Retail price set by the converted enterprise	Decision variable of the capacity-expanding enterprise.
$p_{c}$	Retail price set by the capacity-expanding enterprise	Decision variable of the converted enterprise.
ρ	Consumer preference parameter between products	Captures consumers’ perceived quality, trust, or preference advantage for capacity-expanding enterprises.
μ	Consumer price sensitivity coefficient	Measures how strongly consumers respond to price changes.
c	Unit production cost	Baseline production cost under mature production conditions.
s	Government subsidy coefficient	Policy instrument used to reduce effective production costs and encourage supply.
$\pi_{e}$	Profit of the converted enterprise	Objective function of the leader under decentralized competition.
$\pi_{c}$	Profit of the capacity-expanding enterprise	Objective function of the follower under decentralized competition.
CS	Consumer surplus	Net utility obtained by consumers from purchasing medical supplies.
SW	Social welfare	Overall welfare including consumer surplus, enterprise profits, and subsidy expenditure effects.

#### 3.2.1 Consumer choice and market demand

Assume that consumers’ willingness to purchase masks is represented by v∈(0,1) , which follows a uniform distribution. Each consumer is assumed to purchase at most one unit of the product. The utility functions associated with purchasing from the converted enterprise and the capacity-expanding enterprise are given as shown in Equations 2,3:


$$U_{e}=\nu -\mu p_{e}$$ (2)

$$\begin{array}{c} U_{c}=(1+\rho )\nu -\mu p_{c} \end{array}$$ (3)

Where $p_{e}$ and $p_{c}$ denote the retail prices set by the converted enterprise and the capacity-expanding enterprise, respectively; μ∈(0,1) represents consumers’ perceived preference, β denotes the price sensitivity coefficient ρ∈(0,1) is the preference parameter reflecting consumers’ trust or perceived quality advantage of products supplied by the capacity-expanding enterprise.

Consumers make purchasing decisions based on the principle of utility maximization, determining whether to buy masks and from which type of enterprise to purchase. When a consumer chooses not to purchase, the utility is normalized to zero. Consequently, two critical threshold values exist that characterize consumers’ purchasing behavior. Accordingly, two critical threshold values, $v_{1}$ and $v_{2}$ , divide consumers into three segments: Consumers choose not to purchase masks when their willingness to pay falls below the lower threshold: $U_{e}\leq 0$ and $U_{c}\leq 0$ , $v\in [0,v_{1})$ ; Consumers prefer products supplied by the converted enterprise when their willingness to pay lies between the two thresholds: $U_{e}\geq 0$ and $U_{e}\geq U_{c}$ , $v\in [v_{1},v_{2})$ ; Consumers prefer products supplied by the capacity-expanding enterprise when their willingness to pay exceeds the upper threshold: $U_{c}\geq 0$ and $U_{c}\geq U_{e}$ , $v\in [v_{2},1]$ . The threshold values can be obtained by solving the indifference conditions $U_{e}=0$ and $U_{e}=U_{c}$ , which can be derived from: $v_{1}=\mu p_{e}$ , $v_{2}=\frac{\mu (p_{c}-p_{e})}{\rho }$ .

Accordingly, the market demand shares of the two types of enterprises can be derived. Since consumers’ willingness to pay follows a uniform distribution, the demand share corresponds to the length of the respective valuation interval. The demand share of the converted enterprise is therefore given by: $q_{e}=v_{2}-v_{1}=\frac{\mu (p_{c}-p_{e})}{\rho }-\mu p_{e}$ , Similarly, the demand share of the capacity-expanding enterprise is given by: $q_{c}=1-v_{2}=1-\frac{\mu (p_{c}-p_{e})}{\rho }$ .

To ensure that both enterprises maintain positive market demand shares $q_{e}>0$ , $q_{c}>0$ , certain parameter conditions must be satisfied. Under these feasibility conditions, and incorporating the previously defined total realized market demand D(t)=εQ(t) , the actual market demand fa + ced by the converted enterprise can be expressed as $q_{e}D(t)$ , while that of the capacity-expanding enterprise is given by $q_{c}D(t)$ .

#### 3.2.2 Enterprise profit functions and social welfare

Building upon the enterprise decision-making framework, this subsection further examines firms’ profit functions and the overall composition of social welfare. The following assumptions are introduced. Building upon the enterprise decision-making framework, this subsection further examines firms’ profit functions and the overall composition of social welfare. The following assumptions are introduced:

Let the unit production cost of masks for the capacity-expanding enterprise be denoted by c . Due to production line adjustments and technological adaptation challenges, converted enterprises incur higher production costs. A relative cost coefficient k(k>1) is therefore introduced to capture this difference, such that the effective unit production cost of the converted enterprise is kc . To incentivize production, the government provides a per-unit subsidy s to the capacity-expanding enterprise. For the converted enterprise, the effective per-unit subsidy is adjusted by a relative subsidy coefficient is(i>0) , resulting in a subsidy level of.

Based on the above assumptions, the profit functions of the converted enterprise and the capacity-expanding enterprise can be formulated as follows Equations 4,5:


$$\begin{array}{c} \pi_{e}=(p_{e}-\mathrm{kc}+\text{is})(\frac{\mu p_{c}-\mu p_{e}}{\rho }-\mu p_{e})D(t) \end{array}$$ (4)

$$\begin{array}{c} \pi_{c}=(p_{c}-c+s)(1-\frac{\mu p_{c}-\mu p_{e}}{\rho })D(t) \end{array}$$ (5)

Where $q_{e}$ and $q_{c}$ denote the market demand shares of the converted enterprise and the capacity-expanding enterprise, respectively, which are derived in Section 2.2.1. The term D(t) represents the total realized market demand for masks.

In the context of major public health emergencies, overall social welfare must incorporate not only traditional economic components but also the broader public health and epidemic-control benefits. In this study, social welfare (*SW*) is defined as the aggregate of consumer surplus (*CS*) enterprise profits, Government subsidy expenditures are treated as transfer payments sD(t) and are therefore deducted in the calculation of social welfare, and government subsidy expenditures. The corresponding functional form is expressed as follows Equation 6:


$$\begin{array}{c} \mathrm{SW}=\pi_{e}+\pi_{c}+\mathrm{CS}-\mathrm{sD}(t) \end{array}$$ (6)

Consumer surplus CS measures the total net utility obtained by consumers in the process of purchasing masks. Based on the segmented purchasing behavior driven by heterogeneous consumer preferences, the mathematical formulation of consumer surplus is given as follows Equation 7:


$$\mathrm{CS}=\int_{0}^{v_{1}}0\mathrm{dv}+\int_{v_{1}}^{v_{2}}U_{e}\mathrm{dv}+\int_{v_{2}}^{1}U_{c}\mathrm{dv}$$ (7)

By substituting the utility functions $U_{e}=v-\mu p_{e}$ and $U_{c}=(1+\rho )v-\mu p_{c}$ into the above formulation, we obtain (Equation 8):


$$\begin{array}{c} \mathrm{CS}=\frac{-2p_{e}p_{c}\mu^{2}+\rho +{p_{e}}^{2}\mu^{2}(1+\rho )+{\left(-p_{c}\mu +\rho \right)}^{2}}{2\rho } \end{array}$$ (8)

## 4 Model solution and analysis

### 4.1 Competitive decision-making mode

Under the competitive decision-making scenario, both the converted and capacity-expanding enterprises aim to maximize their respective profits. The capacity-expanding enterprise acts as the market leader, while the converted enterprise serves as the follower. The profit functions of the two enterprises are therefore given as follows Equations 9,10:


$$\begin{array}{c} \pi_{e}=(p_{e}-\mathrm{kc}+\text{is})(\frac{\mu p_{c}-\mu p_{e}}{\rho }-\mu p_{e})D(t) \end{array}$$ (9)

$$\begin{array}{c} \pi_{c}=(p_{c}-c+s)(1-\frac{\mu p_{c}-\mu p_{e}}{\rho })D(t) \end{array}$$ (10)

*Theorem 1:* There exists an optimal pricing strategy for the converted and capacity-expanding enterprises that maximizes their respective profits.

*Proof.* To determine the optimal value of $p_{e}$ , we take the first derivative of $\pi_{e}$ with respect to $p_{e}$ and set it equal to zero, and we can obtain (Equation 11):


$$\begin{array}{c} p_{e}=\frac{p_{c}+(\mathrm{ck}-\text{is})(1+\rho )}{2(1+\rho )} \end{array}$$ (11)

Substituting the reaction function of the converted enterprise into the profit function of the capacity-expanding enterprise. The optimal price of the capacity-expanding enterprise is thus obtained as follows Equation 12:


$${P_{c}}^{*}=\frac{2\rho (1+\rho )-\mathrm{s\mu}(1+i+(2+i)\rho )+\mathrm{c\mu}(1+k+(2+k)\rho )}{2(\mu +2\mu \rho )}$$ (12)

By substituting the above result into the reaction function of the converted enterprise, the optimal price of the converted enterprise can be obtained (Equation 13):


$${p_{e}}^{*}=\frac{1}{4}(2\mathrm{ck}-2\text{is}+\frac{1}{\mu }+\frac{c-s}{1+\rho }+\frac{-1+\mathrm{ck\mu}-\mathrm{is\mu}}{\mu }+2\mu \rho )$$ (13)

By substituting ${p_{c}}^{*}$ and ${p_{e}}^{*}$ into the profit functions of, the optimal profits of the two firms, as well as the optimal consumer surplus and social welfare, can be obtained as follows Equations 14–17:


$$\begin{array}{c} {\pi_{e}}^{*}=\frac{\mathrm{D\mu}{\left((\mathrm{ck}-\text{is})(1+\rho )-\frac{\begin{array}{l} 2\rho (1+\rho )-\mathrm{s\mu}(1+i+(2+i)\rho )\\ +\mathrm{c\mu}(1+k+(2+k)\rho ) \end{array}}{2(\mu +2\mu \rho )}\right)}^{2}}{4\rho (1+\rho )} \end{array}$$ (14)

$$\begin{array}{c} {\pi_{c}}^{*}=\frac{D(t){\left(\begin{array}{l} 2\rho (1+\rho )-\mathrm{s\mu}(-1+i+(-2+i)\rho )\\ +\mathrm{c\mu}(-1+k+(-2+k)\rho ) \end{array}\right)}^{2}}{8\mu \rho (1+\rho )(1+2\rho )} \end{array}$$ (15)

$$\begin{array}{c} \begin{array}{l} CS^{*}=\frac{1}{8\rho }((1+\rho )(M^{2}+4\rho )-\frac{1}{1+2\rho }(M+4\rho )K)\\ +\frac{(1+4\rho )R^{2}}{4(1+\rho ){\left(1+2\rho \right)}^{2}}) \end{array} \end{array}$$ (16)

$$\begin{array}{c} SW^{*}={\pi_{e}}^{*}+{\pi_{c}}^{*}+CS^{*}-\mathrm{sD} \end{array}$$ (17)

*Corollary 1:*

(1) $$\frac{\partial {p_{e}}^{*}}{\partial s}\langle 0,\frac{\partial {p_{c}}^{*}}{\partial s}\langle 0;\frac{\partial {\pi_{e}}^{*}}{\partial s}\rangle 0,\frac{\partial {\pi_{c}}^{*}\;}{\partial s}\rangle 0$$

(2) $$\frac{\partial {p_{e}}^{*}}{\partial k}>0,\frac{\partial {p_{c}}^{*}}{\partial k}>0;\frac{\partial {\pi_{e}}^{*}}{\partial k}<0,\frac{\partial {\pi_{c}}^{*}\;}{\partial s}<0$$

*Corollary 1* (1) indicates that, under the competitive decision-making mode, an increase in government subsidies for both enterprises leads to a reduction in mask prices. Meanwhile, higher subsidy levels increase the optimal profit of the converted enterprise. For the capacity-expanding enterprise, the impact of subsidies depends on the relative subsidy coefficient between the two enterprises. When the subsidy intensity received by the converted enterprise relative to that of the capacity-expanding enterprise falls within a certain range, the optimal profit of the capacity-expanding enterprise increases, the optimal profit of the capacity-expanding enterprise declines. By subsidizing both types of enterprises, the government effectively reduces equilibrium mask prices.

*Corollary 1* (2) indicates that, under the competitive decision-making mode, an increase in the relative subsidy coefficient of the converted enterprise leads to higher mask prices but lower profits for both enterprises. An increase in the relative subsidy coefficient implies that the converted enterprise faces relatively higher production costs. As its marginal production cost rises, the converted enterprise increases its price to compensate for the cost escalation. Anticipating this reaction, the capacity-expanding enterprise, acting as the market leader, adjusts its pricing strategy according to the follower’s reaction function. Consequently, the mask price set by the capacity-expanding enterprise also increases as the relative subsidy coefficient rises. The simultaneous price increases of both enterprises reduce consumers’ willingness to purchase, resulting in a contraction of total market demand. Although higher prices may increase per-unit revenue, the negative impact of reduced demand dominates. Therefore, the optimal profits of the enterprises decline.

### 4.2 Cooperative decision-making mode

Under the cooperative decision-making framework, the converted and capacity-expanding enterprises are regarded as an integrated entity that aims to maximize total joint profits. Production and pricing decisions are therefore determined collectively to achieve the most favorable overall outcome.

The total profit of the integrated enterprise system is defined as Equation 18:


$$\begin{array}{c} \begin{array}{l} \pi_{z}=\pi_{e}+\pi_{c}=(p_{e}-\mathrm{kc}+s)(\frac{\mu p_{c}-\mu p_{e}}{\rho }-\mu p_{e})D\\ +(p_{c}-c+s)(1-\frac{\mu p_{c}-\mu p_{e}}{\rho })D \end{array} \end{array}$$ (18)

*Theorem 2:* Under the cooperative decision-making mode, there exist optimal prices for the converted and capacity-expanding enterprises that maximize their joint profit.

*Proof*: By computing the Hessian matrix of the joint profit function with respect to the decision variables, we obtain:


$$\begin{array}{c} H=(\begin{array}{cc} 2D(-\mu -\frac{\mu }{\rho }) & \frac{2\mathrm{D\mu}}{\rho }\\ \frac{2\mathrm{D\mu}}{\rho } & -\frac{2\mathrm{D\mu}}{\rho } \end{array}) \end{array}$$

The first principal minor of the Hessian matrix is $2D(-\mu -\frac{\mu }{\rho })<0$ , and the second principal minor is $\frac{2D^{2}\mu^{2}(1+\rho )}{\rho^{2}}>0$ . The Hessian matrix is verified to be negative definite based on its leading principal minors, indicating that the joint profit function is concave and possesses a unique optimum. The first-order conditions are derived by differentiating the joint profit function with respect to the pricing variables of the two enterprises and setting $\frac{\partial \pi_{z}}{\partial p_{e}}=0$ , $\frac{\partial \pi_{z}}{\partial p_{c}}=0$ , the optimal prices as follows Equations 19,20:


$$\begin{array}{c} {P_{e}^{z}}^{*}=\frac{1+\mathrm{ck\mu}-\mathrm{is\mu}}{2\mu } \end{array}$$ (19)

$$\begin{array}{c} {P_{c}^{z}}^{*}=\frac{1+\mathrm{c\mu}-\mathrm{s\mu}+\rho }{2\mu } \end{array}$$ (20)

Substituting ${P_{e}^{z}}^{*}$ and ${P_{c}^{z}}^{*}$ into the above expression yields the optimal joint profit $\pi_{z}$ of the converted and capacity-expanding enterprises under the cooperative decision-making mode (Equations 21–23):


$$\begin{array}{c} {\pi_{z}}^{*}=\frac{D(\begin{array}{l} {\left(c(-1+k)+s-\text{is}\right)}^{2}\mu^{2}+\rho \\ +\mu (-2c+2s+{\left(\mathrm{ck}-\text{is}\right)}^{2}\mu )\rho +\rho^{2} \end{array})}{4\mu \rho } \end{array}$$ (21)

$$\begin{array}{c} C{S_{z}}^{*}=\frac{\begin{array}{l} {\left(\text{is}(-1+\mu )-c(-1+k)\mu \right)}^{2}+\rho \\ +(2\text{is}-2\mathrm{c\mu}+{\left(\mathrm{ck}-\text{is}\right)}^{2}\mu^{2})\rho +\rho^{2} \end{array}}{8\rho } \end{array}$$ (22)

$$\begin{array}{c} S{W_{z}}^{*}={\pi_{z}}^{*}+C{S_{z}}^{*}-\mathrm{sD} \end{array}$$ (23)

*Corollary 2:*

(1) $$\frac{\partial {P_{e}^{z}}^{*}}{\partial s}<0,\frac{\partial {P_{c}^{z}}^{*}}{\partial s}\langle 0,\frac{\partial {\pi_{z}}^{*}}{\partial s}\rangle 0$$

(2) $$\frac{\partial {P_{e}^{z}}^{*}}{\partial k}>0,\frac{\partial {P_{c}^{z}}^{*}}{\partial k}=0,\frac{\partial {\pi_{z}}^{*}}{\partial k}>0$$

*Corollary 2* (1) indicates that, under the cooperative decision-making mode, an increase in government subsidies leads to lower mask prices and higher total profits for the integrated enterprise system. Under cooperation, the two enterprises jointly determine optimal prices to maximize collective profit. Government subsidies reduce effective production costs, enabling firms to lower prices to stimulate market demand. The expansion in demand offsets the negative effect of price reductions on revenue, resulting in an overall increase in total profit.

*Corollary 2* (2) shows that, under the cooperative scenario, an increase in the relative cost coefficient of the converted enterprise leads to higher mask prices and greater total joint profits, while the production cost of the capacity-expanding enterprise remains unaffected. When the relative cost of the converted enterprise rises, the integrated system adjusts pricing strategies to mitigate cost pressures. Specifically, the converted enterprise raises its price, while the capacity-expanding enterprise maintains its original cost structure. As a result, consumers shift their purchases toward products supplied by the capacity-expanding enterprise, increasing its market share. This demand reallocation effect enhances overall profitability and leads to an increase in total joint profits.

The equilibrium results reveal that subsidies, relative costs, and consumer preferences affect market outcomes through different mechanisms. Subsidies reduce the effective production cost of enterprises and therefore weaken upward pressure on equilibrium prices. However, the policy effect depends on the subsidy structure. Excessive subsidy bias may alter firms’ strategic pricing responses and lead to inefficient demand allocation. The relative cost coefficient reflects the additional production burden of converted enterprises, including production-line adjustment, technical adaptation, and emergency procurement costs. Consumer preference affects demand allocation by changing consumers’ relative willingness to purchase from capacity-expanding or converted enterprises. Therefore, the equilibrium results reflect not only mathematical optimization, but also the interaction among cost pressure, policy support, consumer trust, and enterprise strategic behavior.

## 5 Numerical analysis

### 5.1 Data processing and construction of epidemic-driven demand

Wuhan epidemic data are used to provide an empirical basis for the epidemic-demand component of the numerical analysis. The Wuhan outbreak is selected because it represents the earliest large-scale COVID-19 public health emergency in China and provides a typical context in which the demand for medical protective supplies increased sharply within a short period. The data are obtained from publicly available daily epidemic reports and consolidated COVID-19 datasets, including official reports released by Hubei and Wuhan health authorities. These sources provide daily information on confirmed cases, recovered cases, and related epidemic indicators during the early stage of the outbreak.

The raw data are first organized into a daily time-series format. The preprocessing procedure includes several steps. First, all date variables are standardized to ensure chronological consistency. Second, duplicate observations from overlapping sources are removed. Third, missing values are checked to maintain the continuity of the daily epidemic series. Fourth, when cumulative confirmed cases are used, they are converted into daily changes where necessary. Fifth, abnormal jumps in the reported series are checked against official announcements, especially when such changes are caused by revised case definitions or reporting corrections. After preprocessing, the cleaned epidemic series is used as the empirical input for the SEIR-based demand construction.

### 5.2 Benchmark parameter setting

The parameter values used in the numerical analysis are reported in Table 3. As this study is primarily a theoretical modeling study supported by real epidemic data, these values are treated as benchmark parameters for mechanism-oriented simulation rather than direct estimates of a specific firm or market transaction. The parameters are selected to ensure consistency with the model assumptions, economic feasibility of equilibrium outcomes, and practical relevance to emergency medical supply production.

**Table 3:** Parameter values.

Parameter	Benchmark value	Basis and real-world relevance
k	1.5	Relative cost coefficient of the converted enterprise. A value greater than 1 reflects higher adjustment, equipment, certification, and operational costs faced by converted enterprises.
i	0.6	Relative subsidy coefficient for the converted enterprise. This captures differentiated government support between converted and capacity-expanding enterprises.
ρ	0.5	Consumer preference or trust parameter. It reflects perceived quality, certification reliability, and trust differences between products from different enterprise types.
μ	0.5	Price sensitivity coefficient. It captures consumers’ response to price changes in emergency medical supply markets.
s	0.3	Per-unit government subsidy. It represents policy support used to encourage production expansion and stabilize emergency supply.

Some parameters are normalized for analytical clarity and cross-scenario comparability. The relative cost coefficient k is set above one to reflect the higher adjustment costs of converted enterprises. The subsidy parameters s and i represent the level and allocation of government support, while the preference parameter captures perceived quality and trust differences between enterprise types. Sensitivity analysis and Monte Carlo simulation are further conducted to test whether the main conclusions remain stable when key parameters vary within plausible ranges. Therefore, the numerical results should be understood as mechanism-oriented simulations rather than direct market-price predictions.

### 5.3 Monte Carlo simulation procedure

To examine the robustness of the numerical results and reduce dependence on a single deterministic parameter setting, this study further conducts a Monte Carlo simulation. The purpose of the simulation is to test whether the main conclusions remain stable when key epidemiological and economic parameters fluctuate within economically meaningful ranges.

For each Monte Carlo run, the epidemiological parameters are randomly drawn from predefined intervals. The transmission rate is sampled from [0.1, 0.5], the incubation transition rate is sampled from [1/14, 1/3], and the recovery rate is sampled from [1/21, 1/7]. These intervals correspond to plausible ranges of transmission intensity, incubation period, and infectious period in an epidemic process. The SEIR system is then solved using numerical integration, and the infectious population trajectory is recorded. In this stage, 100 simulation runs are conducted. The mean infection trajectory and the 95% confidence interval are calculated across all runs and compared with the observed infectious data.

### 5.4 Numerical results and discussion

**Figure 3:**
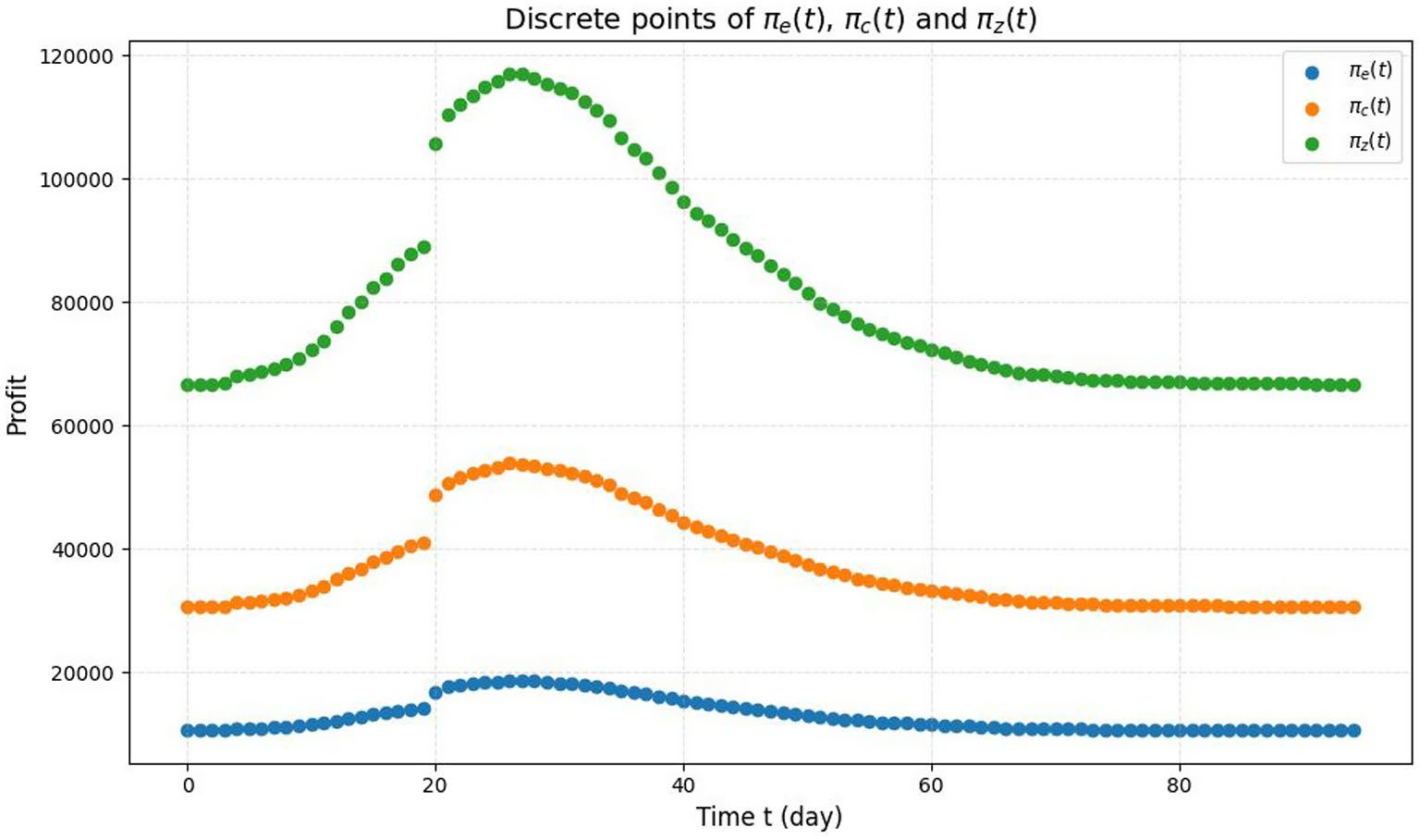
Epidemic evolution chart.

To derive more practical managerial implications and to better understand how variations in government subsidies and relative cost coefficients affect the decisions and profit trends of different supply chain participants under alternative decision-making modes and leadership structures, this study conducts a numerical analysis based on parameter settings adapted from the relevant literature. The selected parameter values are consistent with the assumptions established in the model. The profit evolution trajectory based on Monte Carlo simulation and the time-lag sequence method is shown in Figure 3. This study selected 94 days of data from Wuhan, and the analysis shows that under the given parameter conditions, profits exhibit the typical transmission characteristics of the SEIR model. In the context of a public health emergency, profit evolution is not linear but dynamically adjusted under the combined effects of epidemic impact, demand expansion, and policy regulation. Early-stage profit rises mainly because the spread of the epidemic rapidly increases the demand for materials, prompting enterprises to expand supply, thereby raising profits; mid-term profit reaches its peak, reflecting that the demand peak and the release of enterprise supply capacity are basically synchronous; late-stage profit gradually declines and stabilizes, indicating that as the epidemic alleviates, demand decreases, and market supply gradually recovers, the space for excess profits is compressed, and the system enters a new steady-state equilibrium.

**Figure 4:**
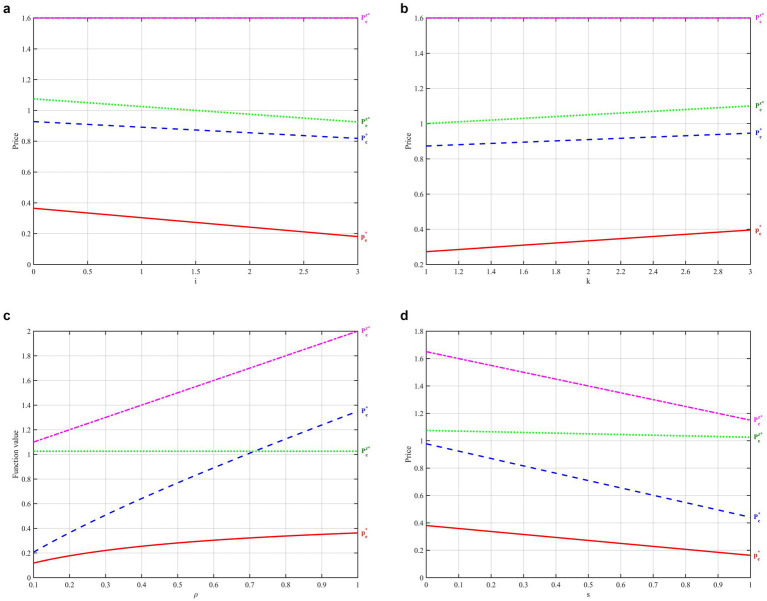
The impact of different parameter changes on the equilibrium price: **(a)** The subsidy level *s* affect the equilibrium price; **(b)** The effect of consumer preference *ρ* on equilibrium prices; **(c)** The effect of the relative subsidy coefficient of the enterprise on the equilibrium price; **(d)** The effect of the relative cost coefficient *k* on the equilibrium price.

The price level is affected by subsidy policies, consumer preferences, and the difference in the comparative advantages of enterprises that switch production and expand production (As show in Figure 4). With the increase of government subsidy level, the overall price variables show a downward trend, indicating that subsidies can effectively alleviate the cost pressure of enterprises and inhibit the rise of market prices. With the enhancement of consumer preference *ρ*, the overall increase of various price variables indicates that the increase in demand-side willingness to pay strengthens the pricing power of enterprises. With the increase of the subsidy coefficient of the enterprises that have changed production compared with the expansion enterprises, the overall price has decreased, indicating that the subsidy tilt toward the enterprises will help enhance their market competitiveness and play a role in stabilizing prices. On the contrary, as the cost coefficient k of the converted enterprises increases relative to the expansion enterprises, the overall price variables increase, reflecting that the higher conversion cost of the converted enterprises will push up the market price through the cost transmission mechanism. In general, government subsidies have obvious price adjustment functions, but their effects still depend on the structure of consumer preferences and the degree to which the relative costs of enterprises are matched with the advantages of subsidies.

**Figure 5:**
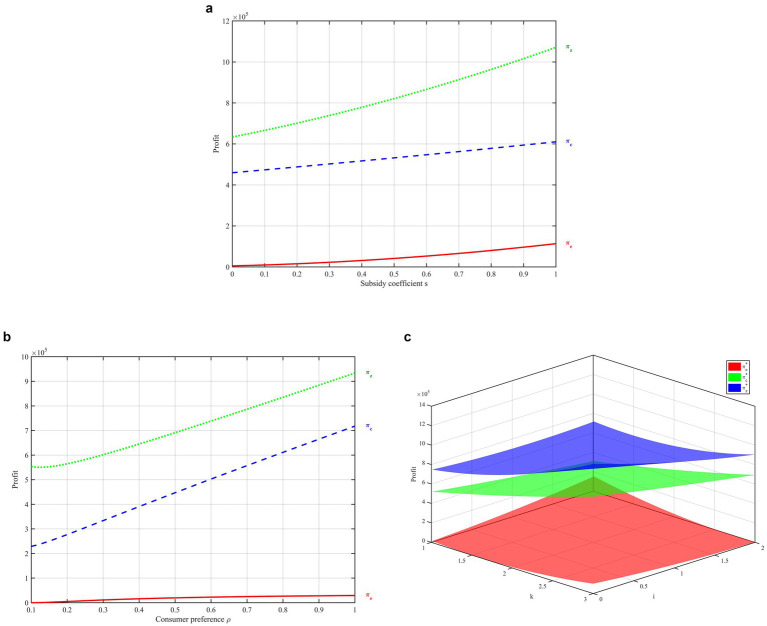
The impact of different parameter changes on the equilibrium profit: **(a)** The subsidy level *s* affect the equilibrium profit; **(b)** the effect of consumer preference ρ on equilibrium profit; **(c)** the equilibrium profit under the relative cost coefficient *k* and the relative subsidy coefficient.

The results show that the changes of different parameters have an impact on the profit of the conversion enterprise, the profit of the expansion enterprise and the total profit of the system, but there are obvious differences in the direction and intensity of the action (As show in Figure 5). With the increase of the subsidy coefficient, the profits are on the rise, of which the total profit of centralized decision-making has the largest increase, followed by the profit of expansion enterprises, and the profit of converted enterprises has also improved simultaneously, indicating that government subsidies can effectively alleviate the cost pressure of enterprises and enhance the enthusiasm of supply. With the strengthening of consumer preference, profits also continue to rise, especially the profits of expansion companies grow faster, indicating that the improvement of demand-side preferences not only expands market capacity, but also further strengthens the profitability of advantageous enterprises. From the perspective of the three-dimensional response surface of k and i , the relative subsidy coefficient and relative cost coefficient of profit have obvious nonlinear characteristics: after the relative subsidy coefficient of the enterprise in production increases, its profit does not increase synchronously, but shows a downward trend. This shows that the increase in subsidies has not been fully translated into net income for enterprises, but has been partially offset by price reductions and revenue compression under the role of optimal pricing and market competition mechanisms.

**Figure 6:**
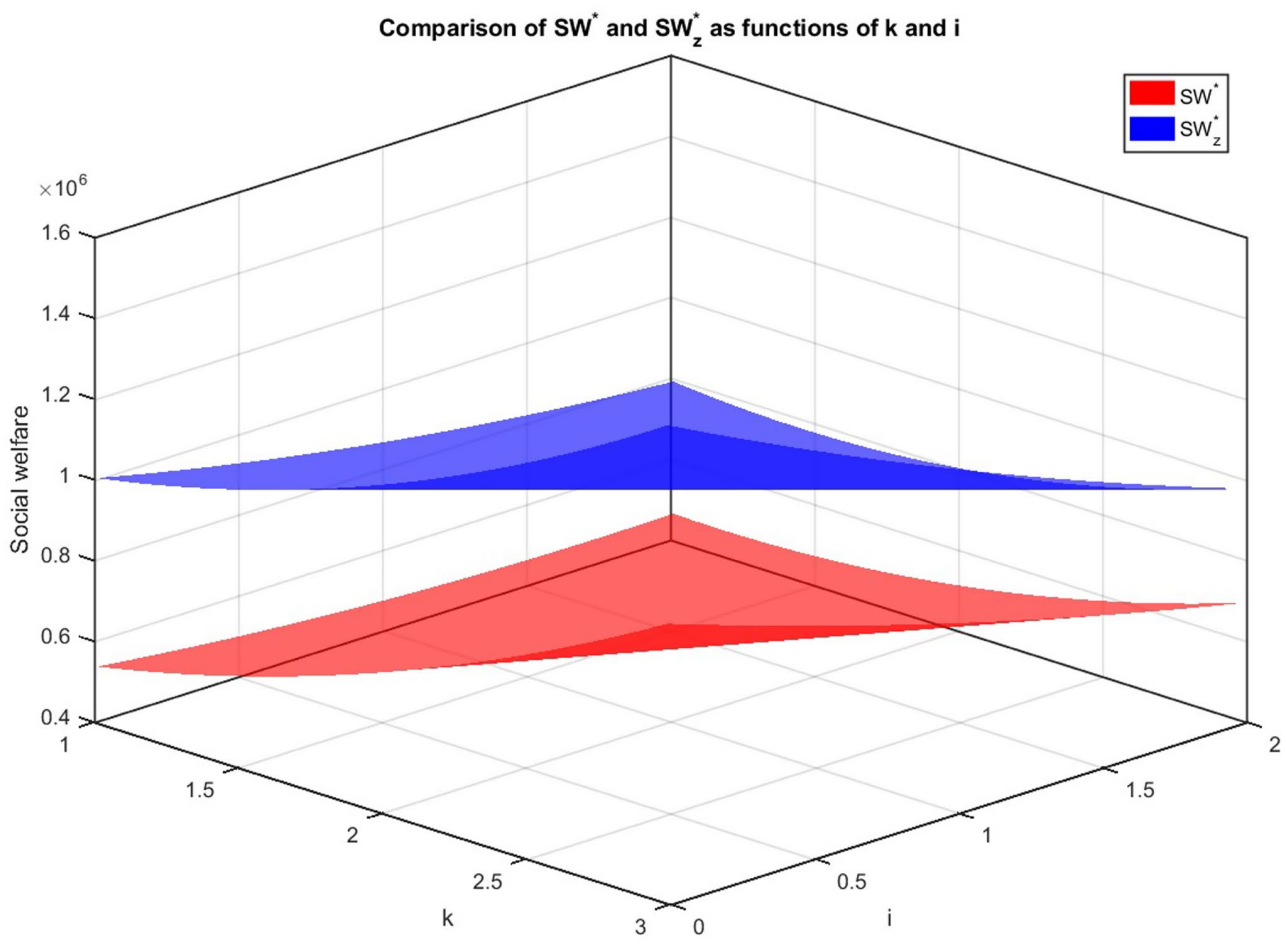
Consumer surplus under the relative cost coefficient *k* and the relative subsidy coefficient.

The Figure 6 shows the three-dimensional change surface of the consumer surplus $CS^{*}$ under decentralized decision-making and the $CS_{z}^{*}$ . Expansion efficiency coefficient k and relative subsidy coefficient i under centralized decision-making. It can be seen that consumer welfare under decentralized decision-making and centralized decision-making has significant interval characteristics. Specifically, in some parameter intervals, $CS^{*}$ . Higher than $CS_{z}^{*}$ , it indicates that when the relative cost and relative subsidy level of the converted enterprises are low, decentralized decision-making is more conducive to maintaining consumer surplus. And with the increase of k and i , $CS_{z}^{*}$ The increase is faster and gradually exceeds $CS^{*}$ , indicating that centralized decision-making can more effectively transform marginal costs and policy support into consumer welfare when the relative cost and relative subsidy of the converted enterprises are high.

**Figure 7:**
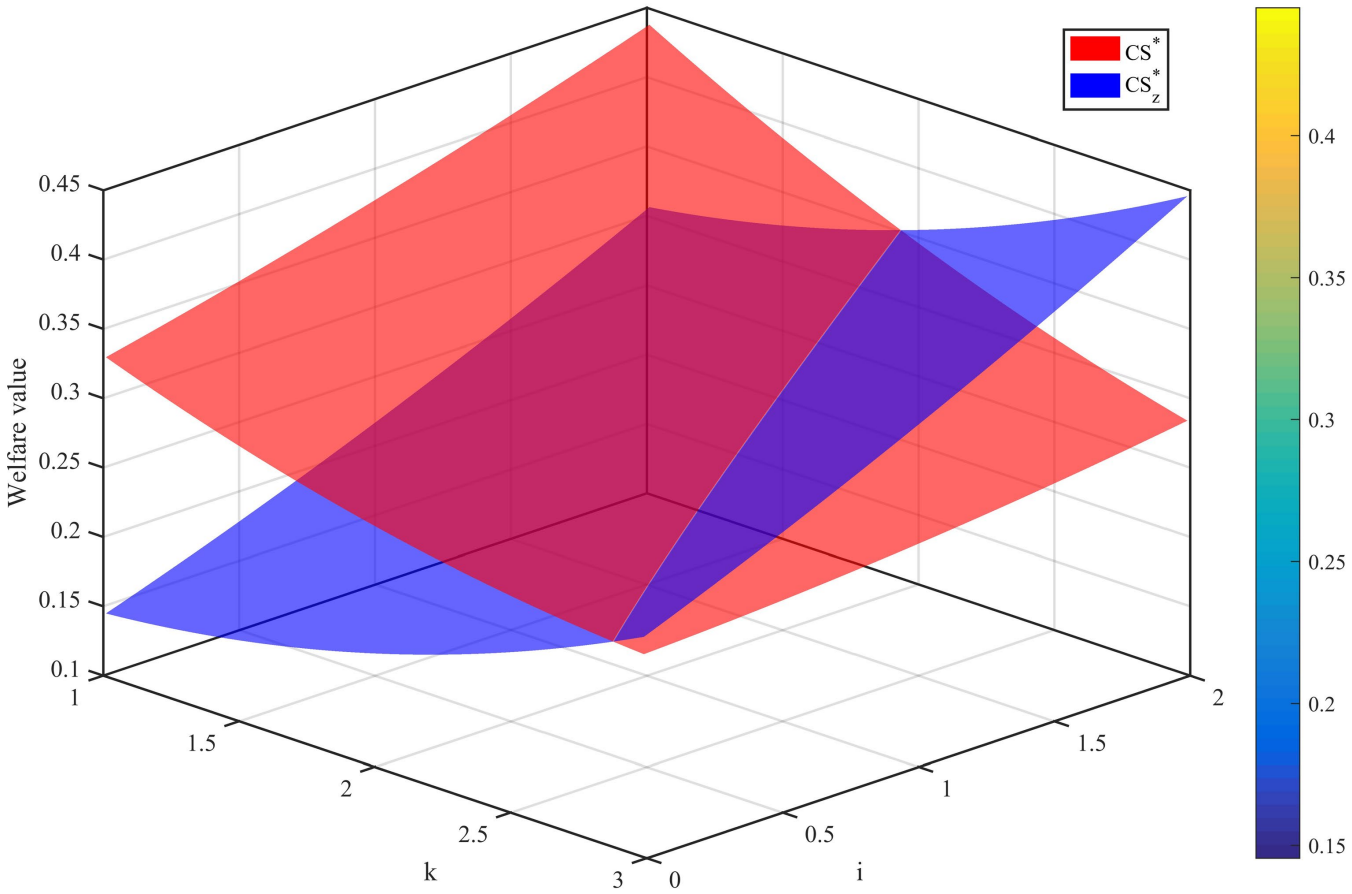
Consumer surplus under the relative cost coefficient *k* and the relative subsidy coefficient.

The social welfare $S{W_{z}}^{*}$ under centralized decision-making is always higher than that of the social welfare $SW^{*}$ . Under decentralized decision-making, indicating that the unified coordination mechanism can effectively improve the efficiency of resource allocation, thereby bringing a higher overall welfare level (As show Figure 7). At the same time, both types of social welfare increased with the increase of the expansion efficiency coefficient k, indicating that efficiency improvement is an important driving factor for welfare growth. The decrease with the increase of the relative subsidy coefficient i indicates that the increase of the bias of the subsidy structure may lead to resource misallocation and the increase of subsidy costs, thereby weakening social welfare. Overall, the graph shows that centralized coordination and efficiency improvement contribute to welfare improvements, while overly biased subsidy arrangements can harm the overall social optimal.

The numerical results show that different parameters affect outcomes through different channels. Subsidies mainly influence prices and profits through the cost-reduction channel. Relative costs affect the competitive position of converted enterprises and may amplify the advantage of capacity-expanding enterprises under decentralized competition. Consumer preference affects the distribution of demand and strengthens the pricing power of enterprises with higher perceived reliability. These results suggest that emergency supply policy should not focus only on production quantity, but also on cost compensation, quality assurance, consumer trust, and coordinated allocation.

## 6 Conclusion

This paper focuses on the problem of consumable medical material security under major public health emergencies, constructs a decision-making analysis framework for the coexistence of converted enterprises and expanding enterprises, and the intervention of government subsidies, and systematically studies the changes of price, profit, consumer surplus and social welfare under different decision-making modes. The results show that the improvement of production expansion efficiency can significantly improve corporate profits and social welfare, and although the subsidy policy has a positive incentive effect, its effect depends on the subsidy structure rather than the simple subsidy scale. When subsidies are excessively tilted toward a certain entity, the policy effect may be weakened through price adjustments and resource misallocation. Further comparison shows that centralized decision-making is more conducive to improving social welfare as a whole, while decentralized decision-making and centralized decision-making show obvious interval differences in consumer surplus. The innovation of this paper mainly lies in the distinction between two heterogeneous entities, conversion enterprises and expansion enterprises, and constructs an integrated analysis framework covering demand shock, corporate decision-making, government subsidies and welfare results, and reveals the influence of factors such as expansion efficiency, relative subsidies and consumer preferences on the profit and welfare transmission mechanism through the comparison of decentralized decision-making and parameter sensitivity analysis.

The findings also provide practical implications for policymakers and emergency management authorities. First, subsidy policies should be designed not only according to subsidy scale, but also according to subsidy structure. A balanced subsidy mechanism is needed because excessive support for a single type of enterprise may lead to resource misallocation and welfare loss. Second, authorities should distinguish between capacity-expanding and converted enterprises. Capacity-expanding enterprises can serve as stable core suppliers, while converted enterprises can provide flexible supplementary capacity when epidemic-driven demand rises sharply. Third, price regulation should be coordinated with subsidy policies. Strict price controls without cost compensation may weaken production incentives, whereas subsidies without price monitoring may fail to protect consumers. Fourth, centralized coordination mechanisms, such as unified procurement, information sharing, and production scheduling, can improve resource allocation efficiency compared with purely decentralized competition. Finally, epidemic data should be integrated into supply planning so that demand forecasts, reserve release, and production incentives can be adjusted dynamically according to infection trends. These implications suggest that emergency medical supply governance should rely on an integrated policy package combining epidemic monitoring, demand forecasting, differentiated subsidies, price guidance, and enterprise coordination.

Overall, the results suggest that emergency medical supply governance should move beyond single-instrument intervention and adopt an integrated policy package combining epidemic monitoring, demand forecasting, differentiated subsidies, price guidance, and enterprise coordination. Such an approach can help stabilize market prices, maintain enterprise participation incentives, protect consumer welfare, and improve the resilience of emergency medical supply systems during major public health emergencies.

## Data Availability

The raw data supporting the conclusions of this article will be made available by the authors, without undue reservation.
